# Biochemical and Computational Studies of the Interaction between a Glucosamine Derivative, NAPA, and the IKK*α* Kinase

**DOI:** 10.3390/ijms22041643

**Published:** 2021-02-06

**Authors:** Mariangela Lopreiato, Samuele Di Cristofano, Rossana Cocchiola, Alessia Mariano, Libera Guerrizio, Roberto Scandurra, Luciana Mosca, Domenico Raimondo, Anna Scotto d’Abusco

**Affiliations:** 1 Department of Biochemical Sciences, Sapienza University of Roma, P.le Aldo Moro 5, 00185 Rome, Italy; mariangela.lopreiato@gmail.com (M.L.); rossana.cocchiola@gmail.com (R.C.); alessia.mariano@uniroma1.it (A.M.); liberagtab3@gmail.com (L.G.); roberto.scandurra@fondazione.uniroma1.it (R.S.); luciana.mosca@uniroma1.it (L.M.); 2 Department of Medicina Sperimentale, Università Magna Graecia, Campus S. Venuta, 88100 Catanzaro, Italy; 3 Department of Molecular Medicine, Sapienza University of Rome, Viale Regina Elena 291, 00161 Rome, Italy; dicristofano.1665448@studenti.uniroma1.it; 4 Clinical Trial Unit, Bambino Gesù Children’s Hospital, IRCSS, P. Sant’Onofrio 4, 00165 Rome, Italy

**Keywords:** IKK, NAPA, osteoarthritis, chondrocytes, UPLC-MS, molecular docking, molecular dynamics

## Abstract

The glucosamine derivative 2-(N-Acetyl)-L-phenylalanylamido-2-deoxy-β-D-glucose (NAPA), was shown to inhibit the kinase activity of IKKα, one of the two catalytic subunits of IKK complex, decreasing the inflammatory status in osteoarthritis chondrocytes. In the present work we have investigated the inhibition mechanism of IKKα by NAPA by combining computational simulations, in vitro assays and Mass Spectrometry (MS) technique. The kinase in vitro assay was conducted using a recombinant IKKα and IKKtide, a 20 amino acid peptide substrate derived from IkBα kinase protein and containing the serine residues Ser32 and Ser36. Phosphorylated peptide production was measured by Ultra Performance Liquid Chromatography coupled with Mass Spectrometry (UPLC-MS), and the atomic interaction between IKKα and NAPA has been studied by molecular docking and Molecular Dynamics (MD) approaches. Here we report that NAPA was able to inhibit the IKKα kinase activity with an IC_50_ of 0.5 mM, to decrease the K_m_ value from 0.337 mM to 0.402 mM and the V_max_ from 0.0257 mM·min${}^{-1}$ to 0.0076 mM·min${}^{-1}$. The computational analyses indicate the region between the KD, ULD and SDD domains of IKKα as the optimal binding site explored by NAPA. Biochemical data indicate that there is a non-significant difference between K_m_ and K_i_ whereas there is a statistically significant difference between the two V_max_ values. This evidence, combined with computational results, consistently indicates that the inhibition is non-competitive, and that the NAPA binding site is different than that of ATP or IKKtide.

## 1. Introduction

The IkB kinase (IKK) complex is involved in several cellular pathways, among them the activation of Nuclear Factor-kB family, (NFkB) which is involved in several aspects of both normal and disease physiology [1]. IKK complex is a high molecular weight multi-subunits complex, which comprises two catalytic subunits, IKKα and IKKβ, and a regulatory subunit, IKKγ/NEMO [2].

The IKKα and IKKβ proteins share high amino acid sequence identity, almost 50%, and their 3D structure presents a kinase catalytic domain (KD) at N-terminal region, a ubiquitin-like domain (ULD) in the middle, followed by an α-helical scaffold/dimerization domain (SDD) and a NEMO-binding domain (NBD) at C-terminal region [2]. Moreover, IKKα also contains a Nuclear Localization Sequence (NLS) [3]. A detailed structural analysis of the IKKβ KD revealed that this domain, when dephosphorylated, shows conformations that are not compatible with its substrates, thus the enzymatic activity is inactive [4,5]. The ULD is required for the activation of kinase activity and, together with SDD, it is involved in the exact positioning of the kinase substrate, which is recruited by NEMO. The interdependence of the three domains is reflected by their intramolecular interactions [5,6]. On the other hand, the IKKβ dimerization depending on SDD is required for binding with NEMO but not for kinase activity after the phosphorylation of the activation loop [5]. To date, the signal transmission leading to IKK activation is still a matter of debate. The presence of kinases that phosphorylate the activation loop or IKK trans-autophosphorylation are both hypothesized [7,8]. In the inactive IKKβ, the dimer shows a very closed structure with the two KD that cannot interact each other, whereas, in activated kinase, the molecules take a more open structure, allowing the KD-KD interactions, likely facilitating the trans-autophosphorylation [5,6]. No information regarding the heterodimer IKKα-IKKβ formation is available. Analyses performed by gel filtration revealed that the complex has a molecular weight of about 700–900 kDa, and equilibrium sedimentation experiments suggest that NEMO has a tetrameric structure. Thus, the hypothesis is that the IKK complex stoichiometry could be IKKα_2_-IKKβ_2_-NEMO_4_ [2]. This organization should bring the catalytic domain of IKKα and IKKβ into proximity [8]. The IKKβ crystal structure analysis suggests that this kinase can form homodimers and tetramers even if to a lesser extent, and that the oligomerization can revert in solution [5,6]. Moreover, other complexes might exist, considering that it has been demonstrated that NEMO can interact with IKKα and IKKβ homodimers [9,10]. Recently, the crystal structure of IKKα has been solved, showing both the protein domains and the supramolecular organization [11]. Like IKKβ, IKKα forms a dimer and each monomer contains KD, ULD and SDD domains. KD shows both inactive and active conformations, ULD serves for interaction with substrates and SDD is involved in the interaction between the monomers, finally, the dimer interfaces of IKKα and IKKβ are highly similar [11].

Globally, the organization of the two kinases is similar, but significant differences can be observed, mainly regarding the orientation of KD with respect to SDD and ULD. In IKKβ, the KD is appropriately positioned with respect to SDD and ULD through the interaction between several residues, Leu389 and Phe390 in ULD, Trp434 and His435 in SDD, and Phe111 and Glu112 in HD [6]. The mutation of these residues in IKKβ reduces the kinase activity. Most of these residues are conserved in IKKα except for Trp434 and Phe111 [11].

The single particle electron cryo-microscopy (cryo-EM) method showed the presence of three predominant conformational states of IKKα, displaying different position of KD, suggesting the mobility of IKKα domains. Moreover, X-ray crystal (PDB: 5EBZ) and cryo-EM structures (PDB codes of IKKα dimer solved by cryo-EM: 5TQW, 5TQX, and 5TQY) together revealed the presence of distinct conformers of IKKα similar to those observed in IKKβ [11]. Finally, Polley et al. observed the formation of further higher order oligomers consisting of three dimers forming an hexamer. The observed interactions were KD-KD, KD-SDD and SDD-SDD [11]. This organization was found only in vitro in X-ray and cryo-EM experiments, whereas the analysis of cellular extracts by Superose size-exclusion chromatography showed the presence of dimers and of an uncharacterized large complex likely containing IKKα, IKKβ and NEMO [11].

The two kinases in vivo show different functions. When IKKβ is associated with NEMO, it activates the canonical pathway of NF-kB [12,13] through the phosphorylation of the isoform alpha of NF-kB inhibitor protein (IkBα). In unstimulated cells, IkB proteins are bound to homo- or hetero-dimers of NF-kB. After pro-inflammatory stimuli, IKKβ phosphorylates IkB proteins, which can be recognized by the ubiquitin ligase machinery, leading to their polyubiquitination and subsequent degradation, allowing NF-kB transcriptional factors to migrate into nuclei and bind specific promoters or enhancer regions of the target genes.

IKKα activates the non-canonical pathway [14], even if IKKα supports the activation of the NF-kB canonical pathway through the phosphorylation of p65 subunit and H3 histone in the nucleus [15]. Moreover, IKKα needs to be associated with the kinase NIK to activate the non-canonical NF-kB pathway. The point of contact corresponds to the sites used by IKKα dimers to form the hexamer, His578 and Tyr580, and Asn408 and Tyr409. The mutation of these sites induces the inhibition of non-canonical pathway [11].

In our lab, we synthesized and studied the effects of a glucosamine-derivative, 2-(N-Acetyl)-L-phenylalanylamido-2-deoxy-β-D-glucose (NAPA) (Figure S1), on human primary chondrocytes coming from patients with Osteoarthritis (OA) [16,17,18,19,20]. The OA is the most common rheumatic disease, associated with ageing, it is characterized by a progressive destruction of the extracellular matrix in cartilage tissue, due to production of pro-inflammatory cytokines in the affected joints. The inflammation leads to the over-production of metalloproteases, causing an imbalance between synthesis and degradation of cartilage [21]. The NF-kB, IKKβ-dependent, canonical pathway has a prominent role in the activation of stress and inflammatory processes involved in OA onset and progression. The IKKα activates the non-canonical pathway, in support of IKKβ action and is also responsible for NF-kB-independent processes, such as the stimulation of several molecules, matrix metalloproteinases (MMPs), transcriptional factors and others, involved in the progression of OA [2,22]. Experiments performed to knockdown IKKα, in three-dimensional culture of articular chondrocytes showed that the absence of this kinase enhanced cell viability, stabilized the extracellular matrix (ECM) and blocked the progression of chondrocytes towards the hypertrophic-like status, which is typical of late OA. Thus, strategies aimed to inhibit IKKα could be very interesting for the treatment of OA. NAPA was found to be effective both to inhibit pro-inflammatory pathways [17,18] and to stimulate ECM components [19,23], and the mechanism of action was based on the inhibition of some kinases, among them IKKα. In our previous work we demonstrated that NAPA was able to inhibit both auto-phosphorylation of IKKα and phosphorylation of IkBα in cell culture of chondrocytes stimulated with the pro-inflammatory cytokine TNFα and in an in vitro assay using [γ^32^P]ATP, whereas very interestingly, it was not able to inhibit IKKβ, showing a specificity of inhibition [20]. In the present manuscript, in order to explore in more detail the interaction between NAPA and IKKα, we performed an in vitro kinase assay and analyzed the results by UPLC-MS. Moreover, we studied the interaction between NAPA and the kinase by means of computational approaches, combining molecular docking experiments with Molecular Dynamics simulations.

## 2. Materials and Methods

### 2.1. UPLC-MS Analyses

LC-MS determination of IKKtide was performed on a Waters Acquity H-Class UPLC system (Waters, Milford, MA, USA), including a quaternary solvent manager (QSM), a sample manager with flow through needle system (FTN), a photodiode array detector (PDA) and a single-quadruple mass detector with electrospray ionization source (ACQUITY QDa). Chromatographic analyses were performed on a Waters C18 BEH column (50 mm × 2.1 mm i.d., 1.7 µm particle size). Solvent A was 0.1% aqueous HCOOH and solvent B was 0.1% HCOOH in CH_3_CN. Flow rate was 0.5 mL/min and column temperature was set at 25 °C.

Elution was performed isocratically for the first minute with 1% solvent B; from min 1 to min 7 solvent B was linearly increased to 70%, then, in 0.5 min solvent B was set at 100% and maintained for 2 min. The column was re-equilibrated with 99% solvent A and 1% solvent B for 3 min before next injection. Standard peptides were IKKtide, a 20 amino acid peptide comprising residues 21 to 41 of IkBα and containing Ser32 and Ser36, pIKKtide, phosphorylated on Ser32, and ppIKKtide, phosphorylated on both Ser32 and Ser36. IKKtide was dissolved in water at a final concentration of 700 µm, pIKKtide and ppIKKtide, were dissolved in Dimethyl sulfoxide (DMSO) at a final concentration of 67 µm. Standard peptides were diluted 1:10 in solvent A immediately before performing the analyses and 20 µL injected through the needle. In the above described chromatographic conditions both peptides have a retention time of ≅3.1 min. Mass spectrometric detection was performed in the positive electrospray ionization mode using nitrogen as nebulizer gas. Analyses were performed in Total Ion Current (TIC) mode in a mass range 500–1200 *m/z*. Capillary voltage was 0.8 kV, cone voltage 8 V, ion source temperature 120 °C and probe temperature 600 °C. Calibration curves were generated with standard peptides synthesized by CliniSciences (CliniSciences S.r.l., Guidonia Montecelio, Italy), by monitoring the H^3+^ ion. These peptides were used to quantify the phosphorylated peptide produced by the enzymatic reaction on IKKtide.

### 2.2. IKKα Kinase Activity Time Course

IKKα kinase activity was investigated performing the reactions at 30 °C for 15′, 30′, 1 h, 2 h, 4 h, and 6 h in a volume of 10 µL in Eppendorf^®^ PCR tubes. IKKα recombinant enzyme (Invitrogen, ThermoFisher Scientific, Waltham, MA, USA) at the final concentration of 0.0607 µg/µL was diluted in a reaction buffer containing 50 mM Tris-HCl, 0.1 M NaCl, 5 mM MgCl_2_ and 1 mM DTT (all purchased from Sigma Aldrich, Co. Saint Louis, MO, USA), in presence of 500 µm Mg^2+^-ATP (Sigma Aldrich) and 0.2 µg/µL IKKtide (Promega Corporation, Madison, WI, USA). The K_m_ value was determined by assaying enzyme activity using the peptide in the range 0.0175–0.175 mM. The amount of IKKα to be used was chosen on the basis of the specific activity declared by the manufacturer. The amount of IKKtide was suggested by the manufacturer’s instructions, finally, the best concentration of ATP was determined in the authors’ laboratory [20]. At the end of incubation time, each sample from all time points was diluted in a 10 µL solution of CH_3_CN/H_2_O (ratio 1:3) to stop the reaction, centrifuged at 14,000 rpm for 10’ to discard any impurities and then ultrapure water was added to the supernatant to reach a final volume of 30 µL, using 20 µL of these for the UPLC/MS analysis.

### 2.3. IKKα Kinase Inhibition

IKKα kinase inhibition by NAPA was investigated as follow: a preliminary incubation step was performed by adding to the reaction buffer the IKKα kinase and NAPA inhibitor at 0.1 mM, 0.5 mM, 1 mM and 2 mM final concentrations and carrying out the reactions at 30 °C for 30’. Then, 500 µm Mg^2+^-ATP (Sigma Aldrich) and 0.2 µg/µL IKKtide (Promega) were added and the samples were incubated at 30 °C for further 1 h. At the end of incubation time, all samples were processed as above described for UPLC analysis. The IC_50_ was calculated using GraphPad Prism software. Moreover, to verify whether the inhibitory effect of NAPA was due to an interaction with the ATP binding site of IKKα enzyme, the reactions were carried out performing a preliminary incubation step by adding in the reaction buffer the IKKα kinase and 0.5 mM NAPA at 30 °C for 30’. Then, Mg^2+^-ATP (Sigma Aldrich), at the following concentrations: 50 µm, 500 µm, 1 mM, 5 mM, plus 0.2 µg/µL IKKtide (Promega) were added, the samples were incubated at 30 °C for further 1 h and then set up for UPLC/MS analysis.

### 2.4. Computational Studies

#### 2.4.1. Molecular Docking and Binding Site Prediction

The three-dimensional coordinates of IKKα hexamer were downloaded from the Protein Data Bank (PDB ID: 5EBZ). The protein chain K, representing IKKα monomeric form, was chosen for subsequent analysis. Atomic coordinates of NAPA were generated by using Avogadro-1.2.0 software [24] and they were subjected to 100 steps of steepest descent and conjugate gradient minimization procedure by using UCSF Chimera software, version 1.13.1 [25].

The AutoDockTools-1.5.7 software [26] was used to prepare IKKα and NAPA. In particular, we used the Python scripts “prepare_receptor4.py” for IKKα and “prepare_ligand4.py” for NAPA. Water molecules were removed, hydrogen atoms and Gasteiger charges were added using the Python scripts “prepare_receptor4.py” for IKKα and “prepare_ligand4.py” for NAPA. All rotatable bonds within the ligand were allowed to rotate freely by setting all possible rotatable bonds and torsions by defining them as active for the compound. Several 10 rotatable bonds, out of the limit of 32 rotatable bonds, was reached. The files were subsequently converted to .pdbqt format. Docking simulations were performed using AutoDock Vina (ADV) [27].

FTMap software tool [28] was used to predict and inspect favorable binding sites on the IKKα crystal structure [11]. FTMap employs a fragment-mapping based approach by using the surface of a target protein, identifying regions of the protein surface that are predicted to form major contributions to the ligand-binding free energy (hot-spots) [28]. FTMap samples billions of positions of small organic molecules used as probes, and it scores the probe poses using a detailed energy expression. The regions that bind several probe clusters are called consensus sites, and the one binding the largest number of probe clusters is considered the main hot-spot. Kozakov et al. established that when using 16 probes, for the mapping, as in this instance, the sites that are known to be druggable invariably contain 16 or more probe clusters (strong “main” hot-spots) [29].

One of the critical parameters for ligand docking is the size of a search space used to identify low-energy binding poses of drug candidates. The docking accuracy of AutoDock Vina is affected by the selection of a search space. The procedure for calculating the optimal docking box size that maximizes the accuracy of binding pose prediction described by Feinstein was used [30]. This method seems to yield better results than the default method not only for experimental pockets, but also for those predicted from protein structures, as in this particular case. The Perl script “eBoxSize-1.1.pl” return the optimal edge length of a cubic docking box, based on the radius of gyration of the ligand to be docked. The calculated dimensions of the search space were 18.529 Å × 18.529 Å × 18.529 Å. AutoDock Vina output is a list of poses ranked by ΔG, the predicted binding energy. We set “num_modes” parameter to 20 in order to obtain the maximum number of poses and “energy_range” to 10.

AutoDock Vina provides a parameter called ‘exhaustiveness’ of individual sampling ‘runs’. This parameter influences the thoroughness of the global search algorithm. Increasing the ‘exhaustiveness’ value decreases the probability of not finding the minimum exponentially (it also increases the computational time linearly). The default exhaustiveness value is 8. To increase the probability of finding the global minimum in our docking experiment, we increase ‘exhaustiveness’ parameter using 11 increasing exhaustiveness levels. We started from 8 and doubling the previous value each time until 8192, in order to increase the probability of the convergence of conformational sampling. We performed 11 runs, one for each exhaustiveness level. To assess result reproducibility, we ran 5 replicate runs for each exhaustiveness level (55 runs), for a total amount of 66 runs. All docking experiments were performed with flexible ligand to enhance the sampling space of IKKα-NAPA interaction. The flexibilization of a maximum of three residues at the same time in the IKKα molecule (receptor molecule) did not yield better results in terms of ligand stability in the binding pocket, as demonstrated by MD simulations (data not shown). Molecular docking results visualization and analysis were performed with UCSF Chimera 1.13.1 [25] and LigPlot+ v2.2 softwares [31].

#### 2.4.2. Molecular Dynamics Simulations

All Molecular Dynamics (MD) simulations were carried out using GROMACS 2019 MD simulation suite [32]. The protein topology was generated using the CHARMM force field (CHARMM36 release, March 2019) [33,34]. The topology parameterization for the ligand was generated with CGenFF software via its web server (https://cgenff.paramchem.org) [35,36]. The complexes were placed in the center of a cubic box and a minimum distance of 1.0 nm between the protein and the box was imposed. TIP3P water model was used for the solvation. Sodium counter ions were added to provide a neutral simulation cell.

Twenty-five thousand steps of steepest descent minimization were performed to relax any steric conflicts. Energy minimization was then followed by atomic relaxation to achieve an equilibrated configuration under the canonical ensemble of constant temperature and volume (NVT) for 5000 ps. The particle mesh Ewald (PME) method [37] was employed to account for the long-range electrostatic interactions, and the LINCS algorithm [38] was used to restrain bond lengths. To maintain a constant temperature of 300 K the velocity rescaling algorithm was used [39]. The production run was performed with leap-frog integrator [39] for a period of 300 ns. We used an integration time step of 2 fs and the coordinates were saved every 10 ps. The production simulation was performed in the canonical ensemble (NVT) according to the work of Bussi G et al. [39]

After MD simulations, the representative structure of the simulated IKKα-NAPA complex was extracted using RMSD conformational clustering algorithm described in [40] implemented in the gmx cluster module of GROMACS through the gromos clustering method, applying a RMSD cut-off of 1.5 Å. Subsequently, the trajectories were analyzed according to the following parameters: Root Mean Square Deviation (RMSD), Root Mean Square Fluctuation (RMSF), hydrophobic contacts, hydrogen bonds and percentage occupancy of hydrogen bonds. Hydrogen bonds between NAPA molecule and IKKα were computed considering the Donor–Acceptor distance cutoff ≤3.5 Å and Donor-H-Acceptor angle cut-off of 30 °C, according to the geometric definition of Luzar and Chandler [41]. In particular for a given H-bond, the higher the rate of occupancy, the greater the number of interactions occurring under 3.5 Å, the higher the level of stability of that H-bond during the MD simulation. RMSD, RMSF and interaction energies were calculated using GROMACS inbuilt modules, as well as the stability of the H-bonds under dynamic conditions. The “readHBmap.py” Python script, developed by R.O. Soares, downloaded from the “Other software” section of the GROMACS website, was used to extract the percentage occupancy of hydrogen bonds from HB Map file (.xpm) generated by gmx hbond routine from GROMACS. Finally, the interaction energies of Coulomb (Coul), Lennard-Jones (LJ) and the sum of Coulomb and Lennard-Jones (Coul + LJ) were calculated using the energy module in GROMACS. GROMACS has the ability to decompose the short-range non-bonded energies via the *energygrps* keyword in the .mdp file. The energy terms of interest are the average short-range Coulombic interaction energy (Coul-SR) and the short-range Lennard–Jones energy (LJ-SR) [42]. Hydrophobic and hydrogen bonding interactions were analyzed with Ligplot+ version 2.2 [31].

## 3. Results and Discussion

### 3.1. IKKα Kinase Assay and Analysis of the Phosphorylated Peptide by UPLC-MS

**Figure 1:**
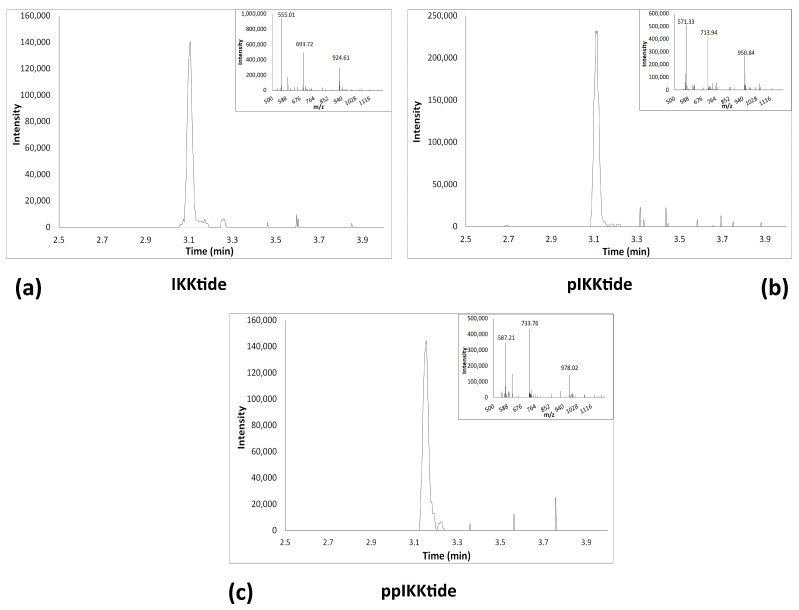
UPLC/MS chromatographic analysis of the three peptides IKKtide, pIKKtide and ppIKKtide. (**a**) unphosphorylated IKKtide peptide (**b**) monophosphorylated pIKKtide peptide on Ser32; (**c**) di-phosphorylated ppIKKtide on Ser32 and Ser36.

The enzymatic activity of IKKα was studied performing an in vitro assay using a recombinant IKKα kinase and a synthetic peptide, IKKtide, containing the serine residues, Ser32 and Ser36, that in vivo are phosphorylated by IKK kinases, ecompassing residues 21–41 of IkBα. We have chosen to use IKKtide as substrate instead of the whole IkBα protein, due to the molecular weight of the peptide that is suitable to be identified by UPLC/MS. Moreover, peptides of approximately 21–23 amino acids have been shown to be good substrates to study the in vitro kinase activity [43,44]. We set up a UPLC/MS method for the identification and quantification of the three peptides: IKKtide, the monophosphorylated form (pIKKtide) on Ser32 and the di-phosphorylated form (ppIKKtide) on Ser32 and Ser36. Standard solutions of synthetic peptides were analyzed by gradient elution using 0.1% formic acid as phase A and acetonitrile containing 0.1% formic acid as phase B. In the described chromatographic conditions IKKtide had a retention time (Rt) of 3.1 min and a *m/z* of 924.61 [M+3H]^3+^ (Figure 1a), whereas, pIKK and ppIKK had similar Rt, and *m/z* of 950.84 [M+3H]^3+^ and 978.02 [M+3H]^3+^, respectively (Figure 1b,c).

**Figure 2:**
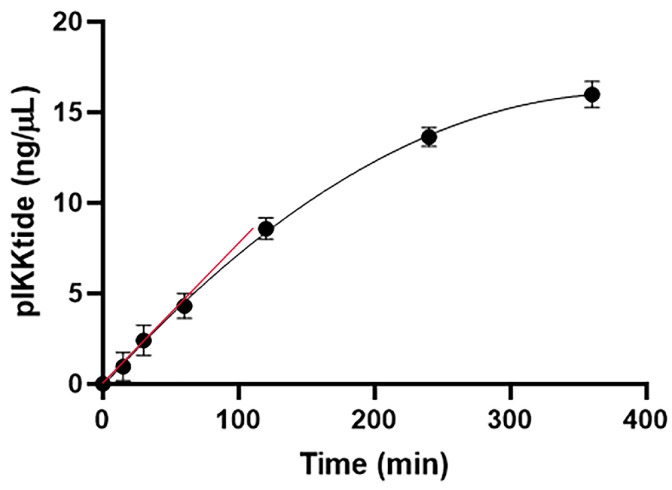
Time-course kinetics of the phosphorylation of IKKtide by IKKα. The kinase assay was performed as described in the text, the results were analyzed by measuring the area of the peak corresponding to the pIKKtide (at *m/z* 951.4 MH3^+^) and the area was converted in ng/µL. The results are reported as mean ± S.E.M. of data obtained by three independent experiments (N = 3).

Next, a time course experiment was performed in order to determine the linear phase of the enzymatic activity. Analyzing the amount of phosphorylated peptide after 15 min, 30 min, 1 h, 2 h, 4 h and 6 h reaction, we found that within the first hour of incubation the enzymatic activity was in the linear phase (Figure 2). Further experiments were performed by stopping the reaction after 1 h. Interestingly, in these reaction conditions, IKKα was able to phosphorylate the IKKtide preferable on one Serine only, at all time points analyzed, considering that we found the pIKKtide was largely exceeding the ppIKKtide amount.

A possible explanation is that the recombinant IKKα we used was unable to phosphorylate both Ser32 and Ser36 in agreement with Kishore et al. who showed that another member of IKK family, the recombinant IKK_i_, was able to phosphorylate only the Ser36 when used in in vitro assay, whereas the immunoprecipitated IKK_i_, from activated cells, was able to phosphorylate both Ser32 and Ser36 on IkBα [43]. These results suggest that in in vitro assays, the recombinant enzyme is less effective compared to enzyme activated in in vivo cells.

Our previous experiments, performed with the whole recombinant IkBα using both the recombinant IKKα and IKKα immunoprecipitated from activated cells, showed that NAPA was able to inhibit both the auto-phosphorylation and the phosphorylation of IkBα [20]. Therefore, it can be hypothesized that the affinity of the enzyme, used in this work, for the peptidic substrate, IKKtide, is lower compared to the whole IkBα protein.

### 3.2. Inhibitory Effect of NAPA on IKKα Kinase Activity

**Figure 3:**
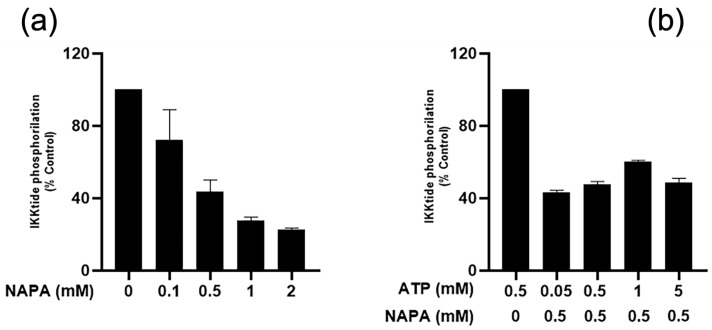
IKKtide phosphorylation inhibition by NAPA. (**a**) The kinase assay was performed as described in the text for 1 h at 30 °C. The NAPA concentrations 0.1 mM, 0.5 mM, 1 mM and 2 mM were used to inhibit the IKKα kinase activity. (**b**) The concentrations of IKKα and NAPA were kept fixed, whereas increasing ATP concentrations were used. The results are reported as mean ± S.E.M. of data obtained by three independent experiments (N = 3). IC_50_ was calculated using using GraphPad Prism software.

To evaluate the IC_50_ of NAPA, different concentrations of NAPA, 2 mM, 1 mM, 0.5 mM and 0.1 mM were pre-incubated with the kinase for 30 min and then 500 µm ATP and 0.2 µg/µL IKKtide as substrate were added and the reaction was stopped after 1 h. In these conditions the NAPA IC_50_ was found to be 0.5 ± 0.086 mM (Figure 3a). This IC_50_ value could seem high, anyway, it has to be considered that a strong inhibition of IKKα could result in a detrimental effect for cells and could display side effects in humans.

Previous studies showed that IKKα-deficient mice displayed serious problems until perinatal death [44,45], whereas, inducible IKKα knockout in adult chondrocytes was not detrimental [46]. IKKα contributes to OA progression through the activation of both canonical and non-canonical NF-kB pathways as well as interferes with extracellular remodeling in cartilage tissue by a kinase-independent activity [22]. Regarding the action of IKKα through the activation of canonical and non-canonical NF-kB pathways, it has been shown that IKKα can shuttle between cytoplasm and nucleus and can promotes the phosphorylation of p65 and Histone H3, which are involved in processes essential for the viability of the cells, for this reason a mild inhibition of IKKα is highly desirable. Considering that, in a previous work, we found that NAPA was able to inhibit the IKKα nuclear migration and in turn the phosporylation of Histone H3 [47], it can be hypothesized that mild effects are effective in the treatment of OA and at the same time safe for individuals. Regarding the effects of NAPA in experiments performed using cellular models, we found that the administration of 0.5 mM showed effectiveness both in inhibiting the inflammatory pathways and in stimulating the production of extracellular matrix components. Likely, the effectiveness of NAPA inside the cells could be due not only to a direct interaction with IKKα but also to a modulation of other intracellular pathways.

To verify whether NAPA inhibited IKKα interacting with the active site of the kinase, we performed the kinase assay using different concentrations of ATP, keeping the concentration of IKKα fixed at 60.7 ng/µL, which was used in all the experiments, and the concentration of NAPA fixed at 0.5 mM, corresponding to its IC_50_. Different kinetic assays were performed using 50 µm, 500 µm, 1 mM, 2 mM and 5 mM ATP. We found that all these concentrations were unable to revert the inhibition of NAPA, suggesting that NAPA did not interact with the ATP binding site of IKKα (Figure 3b).

### 3.3. Kinetics of IKKα and Inhibition by NAPA

**Figure 4:**
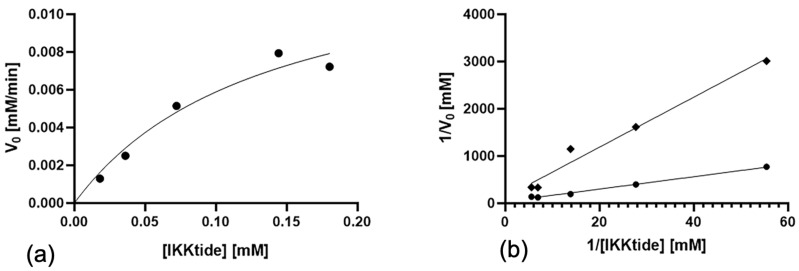
Michaelis-Menten and Lineweaver-Burk fitting. (**a**) several concentrations (ranging from 0.0175 mM to 0.175 mM of IKKtide were analyzed in the in vitro kinase assay as described in the text, to calculate the Km and the Vmax; (**b**) to calculate the Ki, same concentration of IKKtide were used in the in vitro assay in absence (circles) and in presence (squares) of 0.5 mM NAPA (corresponding to IC_50_). K_m_, V_max_ and K_i_ were calculated with GraphPad Prism software (N = 3).

To confirm the hypothesis that the inhibition of NAPA on IKKα activity was non-competitive, we performed a kinetic assay using a fixed amount of enzyme and increasing concentrations of substrate, IKKtide, starting from 0.0175 mM to 0.175 mM. The resulting kinetic curves were fitted with Michaelis-Menten model, finding a K_m_ of 0.337 ± 0.193 mM and a V_max_ of 0.0257 ± 0.00760 mM·min${}^{-1}$ (Figure 4a). The K_m_ we determined in our experiments is higher than K_m_ reported in a previous manuscript (0.0237 ± 0.00150 mM) [44] where the same peptide was used as substrate. This apparent discrepancy could be attributed to the fact that in the paper of Huynh et al. the IKKα used was a homodimer, whereas we used a commercial IKKα, which is a monomer. Moreover, we conducted the same experiments in the presence of different concentrations of NAPA, and the resulting kinetic curves were plotted with Lineweaver-Burk, obtaining a K_i_ 0.402 ± 0.0416 mM and V_max_ 0.0076 ± 0.000552 mM·min${}^{-1}$ (Figure 4b). The difference between K_m_ and K_i_ is not statistically significant. In contrast, we found a statistically significant difference between the V_max_ values obtained in absence or in the presence of NAPA (*p* value = 0.021), strongly suggesting that the inhibition could be non-competitive. NAPA is a small and safe molecule. It is a glucosamine-derivative, showing a N-Acetyl phenylalanine coupled to aminic group of the sugar (see Figure S1). The inhibition of kinase activity of IKKα by NAPA makes it a very appealing molecule. The ATP binding site is conserved among kinases, and considering that phosphorylation has a central role in biological regulation of intracellular pathways, the non-specific inhibition of those processes is absolutely undesirable. Our findings, demonstrating that NAPA does not bind to the ATP binding site, show that this molecule is extremely interesting and particularly suitable for long-term treatments, such as those required for OA.

### 3.4. Computational Studies

#### 3.4.1. Identification of Putative NAPA Binding Sites on IKK*α* and Their Characterization

To identify the NAPA binding sites of IKKα suitable for the docking studies, we first used a computational fragment screening approach (FTMap) [28]. FTMap analysis, carried out in both monomeric (chain K) and dimeric (chain K and L) forms of IKKα, identified the same region as best “drugguble” hot-spot (hereafter defined as Binding Site 1, BS1), potentially involved in interaction with the NAPA molecule. All the results obtained by using FTMap approach are shown in Figure S2. Using the cluster of probes in the BS1 (probe clusters in the green circle reported in Figure S2), a purely geometric, non mass-weighted centroid was defined for the IKKα protein structure. The coordinates of the centroid were used to define the center of the searching space in the docking experiment, i.e., the docking box.

**Figure 5:**
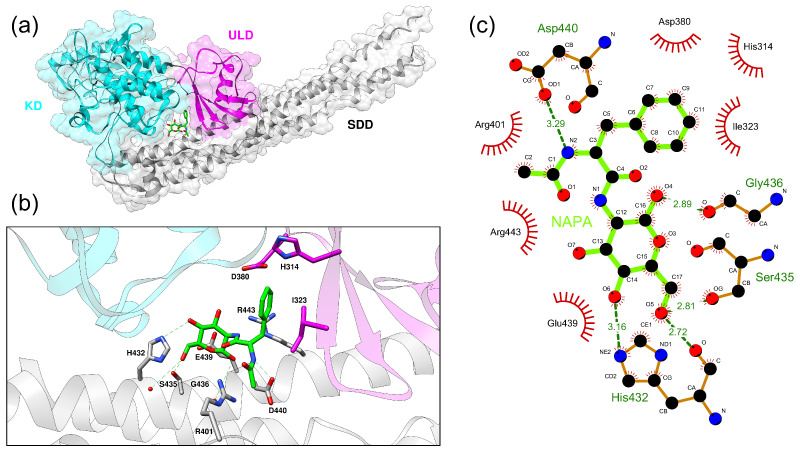
IKKα-NAPA complex top-ranked pose of the focused molecular docking experiment. (**a**) IKKα is depicted as cartoon. NAPA molecule is represented in stick model (green). KD, ULD and SDD domains are colored in cyan, magenta and gray respectively. (**b**) Interacting residues are represented in stick model. Dashed red lines indicate hydrogen bonds. (**c**) IKKα-NAPA non-covalent interactions. Dashed green lines indicate hydrogen bonds, red lines indicate hydrophobic interactions.

BS1 takes place in a cavity between the KD, ULD and SDD domains (Figure 5) and it is defined by the following residues: Lys117, Glu118, Asn262, Ser263, Cys265, Hys314, Asp380, Hys432, Arg443 (UNIPROT numbering). We also compared FTMap binding sites prediction performed on IKKα with that performed on IKKβ: although the two proteins are very similar (almost 60% sequence identity), they present a different position of “druggable” binding sites (Figure S4).

Subsequently, we obtained a more accurate picture of the binding modalities of NAPA with IKKα by means of a molecular docking experiment. In our docking experiments we focused on the BS1 identified by the FTMap algorithm that is far from the kinase catalytic site. Therefore, we excluded NAPA from the kinase catalytic site (third position in the FTMap output ranking) in docking experiment by running a local molecular docking. We centered the search space of the docking experiment in BS1 and the docking analyses were performed using the Autodock Vina program (ADV) [27] using the protocol described in the Methods section. Figure 5 shows the best docked conformation (top-ranked pose) of NAPA in the BS1 cavity based on the highest ADV score reached at the convergence of conformational sampling. We also tried a docking experiment on the best pocket of IKKβ but the ADV score did not reach convergence, perhaps because the best scoring binding site on the IKKβ surface is too small to accommodate NAPA in a plausible conformation. We report all the result obtained by FTMap in the Figure S4. This could clearly correlate with different behavior of NAPA on IKKα over IKKβ, we already observed in our previous work.

This pose was take into consideration for a first ligand–receptor interactions analysis that is shown in Figure 5b,c. A close analysis of the molecular environment of the binding site reveals that the closest amino acid residues interacting with NAPA are: His432, Ser435, Gly436, Asp440 via H-bonds (green lines in Figure 5b), and Asp380, His314, Ile323, Glu439, Arg401 and Arg443 by means of hydrophobic interactions (red lines Figure 5b). MD was used to refine the selected binding pose of NAPA in BS1.

#### 3.4.2. IKK*α*-NAPA Complex Molecular Dynamics Simulation

**Figure 6:**
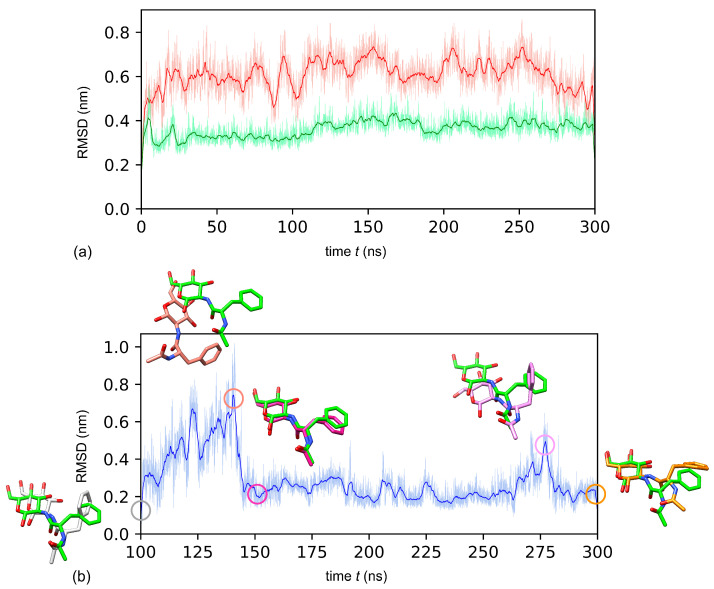
RMSD trajectory of IKKα and NAPA. (**a**) IKKα presents higher backbone RMSD values in the NAPA-unbound form (6.1 ± 0.8 Å) compared with the NAPA-bound form (red line) (IKKα-NAPA complex), (green line) (3.6 ± 0.4 Å). (**b**) RMSD of the ligand heavy atoms evolution of the NAPA molecule alone (2.38 ± 0.65 Å). Bold lines represents running averages, while light lines indicates individual time steps spaced 10 ps apart. Overlay of the representative NAPA pose selected from the MD trajectory (reported in green) with important intermediates along the trajectory are depicted in stick representation (see the main text for further details). Overlay of the initial and final poses from the MD simulation is reported in Figure S3.

To increase the accuracy of the NAPA-IKKα complex, MD simulation was employed, taking into account solvent effects, as well as protein and ligand dynamics, potentially correcting some of the deficiencies associated with the docking protocol or scoring function. MD simulations were performed up to 300 ns to analyze the conformational behaviour of the NAPA molecule, and the complex formed with IKKα protein in the solvated model system with the goal of optimizing the ligand orientation. To explore dynamic perturbation in the conformation of the IKKα-NAPA complex, simulations were carried out for two different systems: (i) IKKα in NAPA-unbound form, (ii) IKKα-NAPA complex (NAPA-bound form). Assessment of the MD data was based on changes in the position of the ligand. The Root Mean Square Deviation (RMSD) from the protein backbone through the 300 ns trajectory—in NAPA-unbound form, in complex with NAPA and for the ligand alone are shown in Figure 6a,b.

As shown in Figure 6a IKKα presents higher backbone RMSD values in the NAPA-unbound form (red line) (6.1 ± 0.8 Å) compared with the NAPA-bound form (IKKα-NAPA complex) (green line) (3.6 ± 0.4 Å), thus suggesting that structural fluctuations of IKKα monomer were found less pronounced in the presence of NAPA.

**Figure 7:**
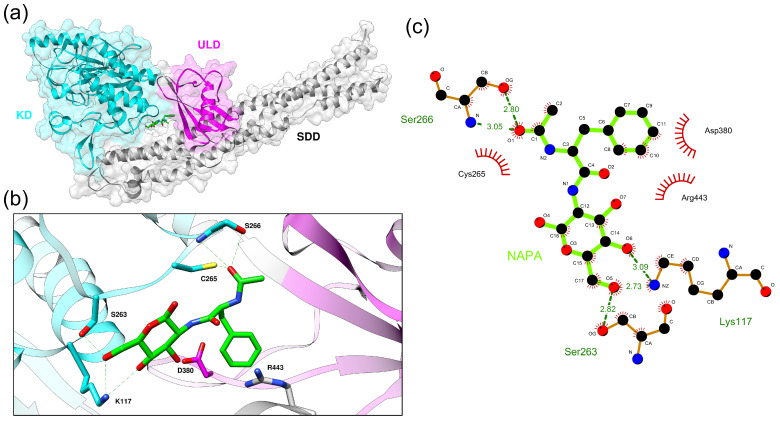
Representative structural model of the IKKα-NAPA complex after MD simulation refinement. (**a**) IKKα is depicted as cartoon. NAPA molecule is represented in stick model (green). KD, ULD, and SDD domains are colored in cyan, magenta, and gray, respectively. (**b**) Interacting residues are represented in stick model. Dashed red lines indicate hydrogen bonds. (**c**) IKKα-NAPA non-covalent interactions. Dashed green lines indicate hydrogen bonds, red lines indicate hydrophobic interactions.

To investigate whether a global conformational heterogeneity in the IKKα-NAPA complex could be detected during MD simulations, clustering analysis was performed using all the frames. In particular NAPA molecule was used for clustering and we selected the central structure of the largest cluster as NAPA representative pose along the trajectory. GROMOS clustering algorithm was able to select four clusters. First cluster comprised the 97% of the frames, while the second, the third and the fourth clusters comprised the 2.3%, 0.6% and 0.12% of the frames respectively. To choose a reasonable RMSD cut-off, we varied the RMSD cut-off between 1 Å (the default value) to 2 Å in steps of 0.5 and performed clustering analysis for each RMSD cut-off value. We choose RMSD cut-off of 1.5 Å value in order to balance between the number of clusters retrieved and the number of frames contained in each cluster. The centroid of the most populated cluster has been reported in Figure 7a–c.

We used the pose of NAPA at 100 ns of MD, when it reached equilibrium, (gray circle in Figure 6b) in order to evaluate RMSD fluctuations for the remaining part of the trajectory (Figure 6b). The fluctuation region we observed in the trajectory from 125 ns to 140 ns (salmon circle marks the peak in Figure 6b), was caused by the movement of NAPA slightly away from the deeper region of the cavity, even though after 140 ns NAPA accommodates again in the cavity (purple circle in Figure 6b). The second minor peak at 275 ns is largely due to a transient movement of the benzyl group (see pink and orange circles in Figure 6b).

In particular, when we analyzed conformational behaviour of NAPA atomic portions along MD simulation, we noted that after 1 ns while the N-acetyl group maintains his orientation, the glucosamine rotates about 90 degrees, compared to the initial docked pose and this new orientation is maintained along the whole simulation. At 100 ns the orientation of NAPA is very similar to the orientation of NAPA extracted from the most representative cluster (Figure 6b). At this point the N-acetyl group of NAPA has switched its orientation and it is stabilized by the contacts with Ser266 and Cys265 in the KD (representing the “roof” of the cavity) (Figure 7b). Moreover, the NAPA benzyl group accommodates deeper in the cavity, compared to the starting conformation of the docked complex, thus establishing contacts with Asp380 and Arg443 (Figure 7b,c): these residues were not fully accessible in NAPA-unbound form of IKKα, i.e., MD simulation equilibrates the system to achieve a stable conformation of NAPA after 100 ns, following a conformational variation of the cavity to better accommodate NAPA in an induced-fit fashion.

**Figure 8:**
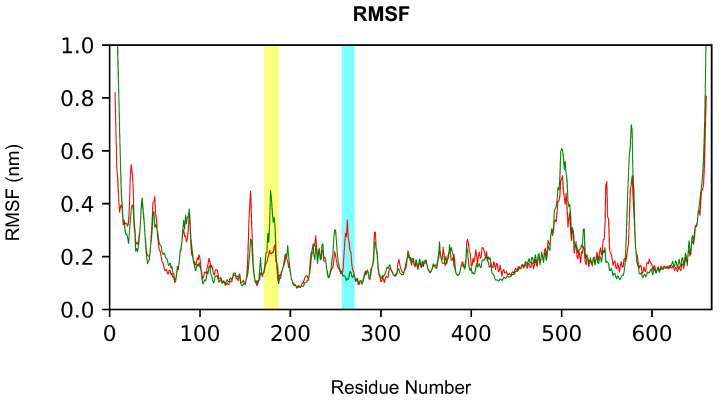
Root mean square fluctuations (RMSF) of NAPA-unbound form (red) and NAPA-bound (green) forms of IKKα. The portions with fewer fluctuations in the bound state are highlighted in cyan and yellow. RMSFs were computed for all atoms in the 300 ns MD trajectories.

We further assessed residues contributing to receptor-ligand complex structural fluctuations by analyzing Root Mean Square Fluctuations (RMSFs) of each IKKα residue. Apart from the N- and C-terminal regions showing high fluctuations, since they tend to be more exposed on the surface with greater mobility, we detected similar global fluctuations in both systems. However, few specific residues showed a significant difference between IKKα NAPA-unbound form and NAPA-bound form forms (Figure 8). In particular, amino acid residues range from 260 to 267 (highlighted in cyan in Figure 8), involved in the binding of NAPA, exhibited a higher RMSF value for IKKα NAPA-unbound form (red line) than NAPA-bound form (green line). We think that presence of the ligand could be able to stabilize side chains fluctuation through the H-bonds network created with Asn262, Ser263, and Ser266 (Figure 7). More interestingly, we observed that the activation loop (residues 176–180 of IKKα) exhibits a higher fluctuation when NAPA is bound to BS1 (Figure 8, region highlighted in yellow), compared to NAPA-unbound form of IKKα (red line). This aspect related to the presence of NAPA in the BS1 could partially describe how NAPA may allosterically modulate conformational change of IKKα, inducing inhibition of IKKα activity.

#### 3.4.3. Hydrogen Bond Analysis of IKK*α*/NAPA Complex

**Figure 9:**
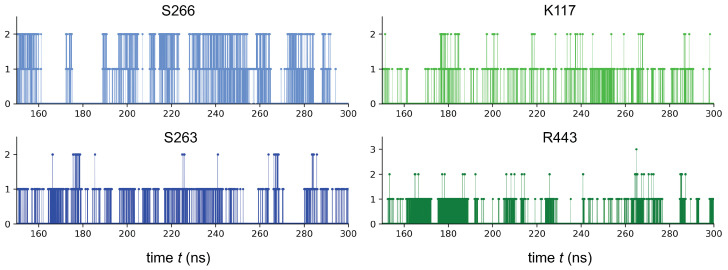
Evolution during the MD simulation (from 150 to 300 ns) of H-bonding network found in IKKα-NAPA complex. We reported H-bond evolution for residues of BS1 showing the top 5 occupancies.

Hydrogen bonds (H-bonds) are facilitators in protein-ligand systems to stabilize the ligand in the binding pocket [48]. Therefore, we investigated the stability of the H-bond network in the IKKα/NAPA complex under dynamic conditions. First, we calculated the number of H-bonds between NAPA and IKKα throughout the MD. The maximum number of H-bonds reached during the simulation between NAPA and IKKα was ten. Subsequently, we analyzed the number of H-bonds and their stability during the MD simulations from 150 ns to 300 ns. We evaluated H-bond occupancy parameter, generally used to study the evolution of the interaction between ligand and receptor (see methods for details). In Table 1 we reported the top 5 occupancies for the hydrogen bonds of NAPA with BS1 region. On the other hand, in Figure 9 we show the evolution of the H-bond number between NAPA and IKKα residues showing the highest H-bonds percentage occupancy (from 150 to 300 ns of the MD trajectory). These analyses allowed us to define a clear dynamic H-bond network between NAPA and IKKα. Thus, a site-directed mutagenesis towards these residues could provide an experimental insight regarding the NAPA binding mode to the IKKα protein, strengthening the hypothesis derived from our simulation.

Finally, we calculated a “decomposition” of the NAPA binding energy into the Coulomb and the VdW contributions [42,49]. This quantity should not, however, be confused with a “binding energy” or a free energy of any sort. It is simply a decomposition of the potential energy of the system, including only nonbonded terms between the selected atom groups. The IKKα-NAPA complex presents values of Short-range interaction energy of Coulomb (Coul) equal to −128.705 ± 52.3 kJ·mol${}^{-1}$, Lennard-Jones (LJ) energy equal to −113.45 ± 17.2 kJ·mol${}^{-1}$ and and sum Coul + LJ equal to −241.850 ± 55.0 kJ·mol${}^{-1}$.

## 4. Conclusions

NAPA has been shown to be effective in counteracting inflammatory pathways and stimulating the production of ECM components in in vitro and in vivo OA models [17,18,19,20,23,47,51]. Its mechanism of action is mainly based on the inhibition of the kinase activity, in particular we found that NAPA was able to inhibit IKKα, whereas it was not able to inhibit IKKβ, showing a specific inhibitory activity. In the present work, we analyzed the mechanism of inhibition of IKKα by NAPA and the putative interaction mechanism between the two molecules. We found that NAPA inhibits this kinase through an allosteric mechanism, considering that increasing amounts of ATP were not able to revert the NAPA inhibition. Computational analyses indicate a plausible binding site, taking place in a cavity between the KD, ULD and SDD domains of IKKα, far from the ATP binding site and catalytic residue. We were able to predict and explore the putative binding pocket, BS1, by means of “druggable” hot-spot searching alghorithm (FTMap) and molecular docking approach, complementing these two analyses by running MD simulations in water of IKKα monomer in NAPA-bound and NAPA-unbound states. We applied this computational workflow in order to increase the accuracy of the NAPA-IKKα complex, taking into account solvent effects, as well as protein and ligand dynamics. In this way we potentially corrected some of the deficiencies associated with the molecular docking protocol or scoring function. We would like to underline how, thanks to the rapid development of both computer hardware, software, and algorithms, as a supplement to experiments, important tools such as target/ligand databases, protein modelling, molecular docking methods, etc., are more and more useful, day after day, for identifying drug binding sites and elucidating drug action mechanisms, just as we did with IKKα/NAPA predicted complex. In addition, biomolecular simulations allow for investigations of both structural and thermodynamic features of target proteins/ligand/complexes on different levels. Of course, the majority of all the approaches are methodologies that fit experimental data and the performance of these methods varies greatly with target macromolecules, available experimental data, and available resources. Therefore, despite the absolute need for subsequent experimental validation, the in silico approach we used in this work might furnish, as a first step, a possible effective picture of the molecular details involved in the biochemical processes related to IKKα inhibition mechanism by NAPA molecule.

Pharmacological therapy is in search of small molecules able to inhibit kinases, in which activation is involved in onset and progression of several diseases. The majority of small molecules approved for pharmacological therapy are type I or type II inhibitors, thus targeting the ATP-binding pocket of kinases. Due to the sequential and structural similarity among ATP pockets, inhibitors that bind these pockets are poorly selective for a specific kinase. On the contrary, allosteric inhibitors, targeting kinases in sites out the highly conserved ATP pockets are much more specific and thus much more interesting as therapeutic approach [52]. The finding that NAPA inhibits IKKα by an allosteric mechanism makes this molecule particularly interesting for the treatment of OA, which is a disease that has an early onset and has a chronic nature.

## Figures and Tables

**Table 1:** Occupancy rates of the stable interactions belonging to the H-bonding network found in IKKα-NAPA complex during the MD simulation, starting from 150 ns to 300 ns. Hydrogen bonds with values of percentage occupancy higher than 10% were considered as a significant non-covalent interaction for the molecular stabilization of the IKKα-NAPA complex [50].

Donor (Atom *)	Acceptor (Atom)	% Occupancy
Ser266 (HN)	NAPA (O1)	43.7
Ser266 (HG1)	NAPA (O1)	17.6
Ser263 (HG1)	NAPA (O5)	15.2
Lys117 (HZ1)	NAPA (O6)	11.1
Arg443 (H11)	NAPA (O2)	10.0

* Atom names are based on GROMACS nomenclature.

## Data Availability

The data presented in this study are available on request from the corresponding author.

## Supplementary Materials

The following are available online at https://www.mdpi.com/1422-0067/22/4/1643/s1. Figure S1: 2D representation of the NAPA molecule. Figure S2: Computational solvent mapping of IKKα using FTMap. Figure S3: Overlay of the initial (yellow) and final (green) poses from the MD simulation. Figure S4: Comparison of FTMap binding sites prediction performed on IKKα over IKKβ.
