# Supplementary material for: Biochemical and Computational Studies of the Interaction between a Glucosamine Derivative, NAPA, and the IKKα Kinase
Source: Int J Mol Sci. 2021 Feb 6;22(4):1643. doi: 10.3390/ijms22041643 (PMC7915277; doi:10.3390/ijms22041643)
Supplement: Supplementary file 1 [file ijms-22-01643-s001.pdf]

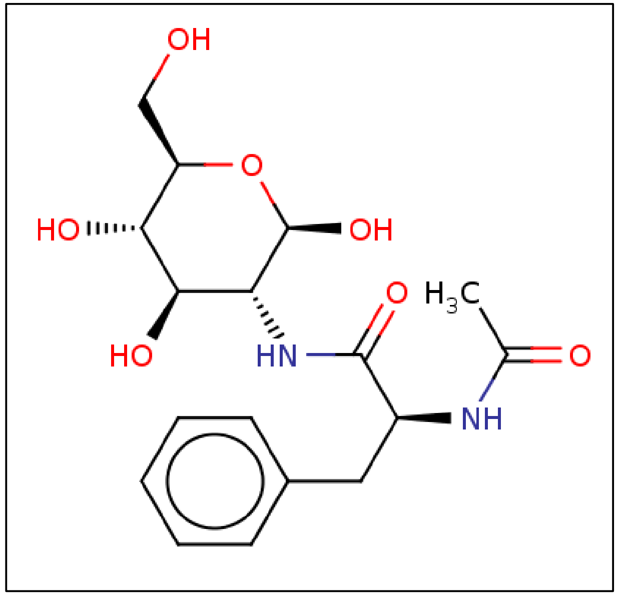

Figure S1. 2D representation of The glucosamine derivative 2-(N-Acetyl)-L-phenylalanylamido-2-deoxy-β-D-glucose1; (NAPA).

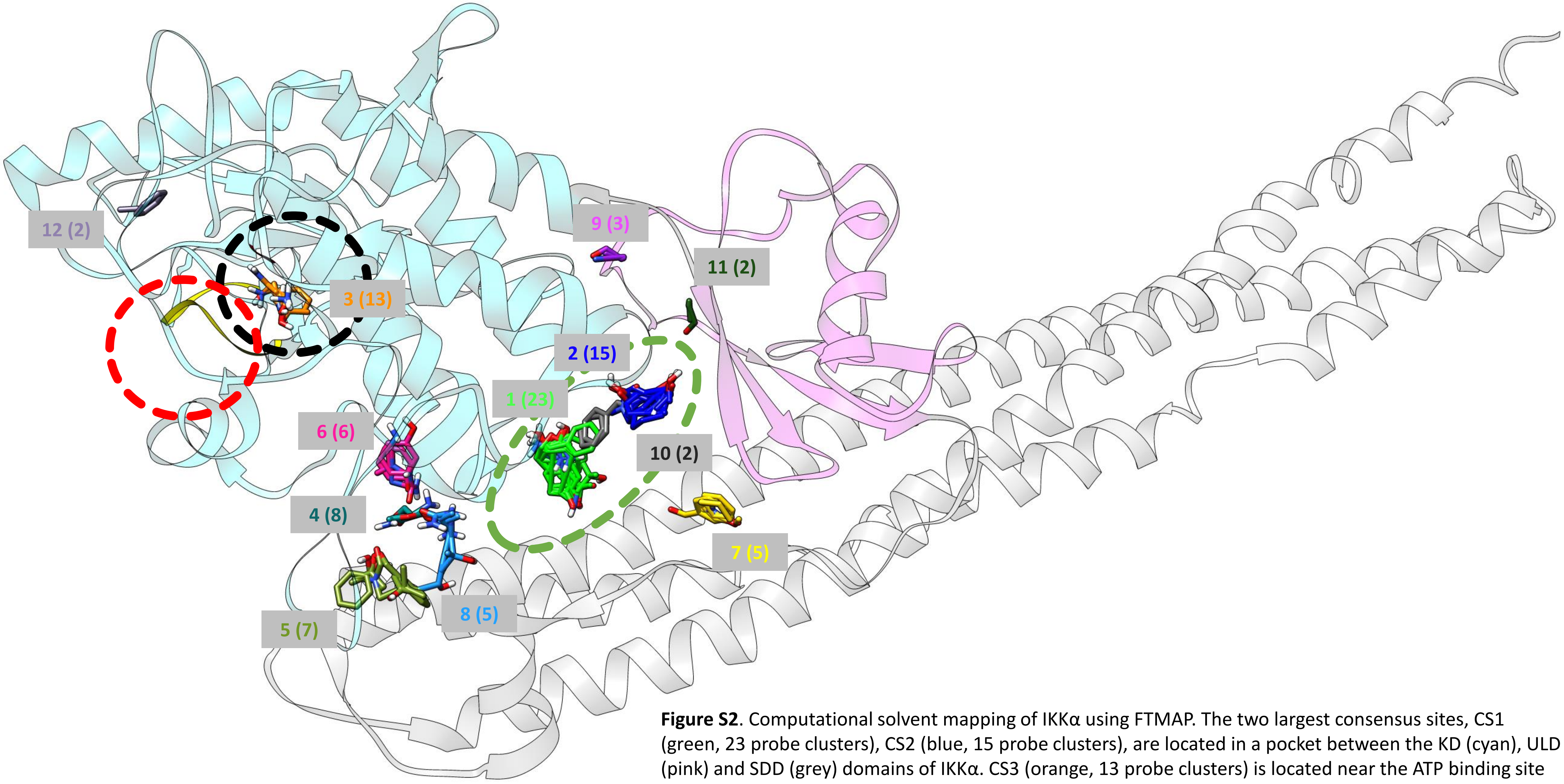

Figure S2. Computational solvent mapping of IKKα using FTMAP. The two largest consensus sites, CS1 (green, 23 probe clusters), CS2 (blue, 15 probe clusters), are located in a pocket between the KD (cyan), ULD (pink) and SDD (grey) domains of IKKα. CS3 (orange, 13 probe clusters) is located near the ATP binding site (black circle) and the activation loop (red circle).

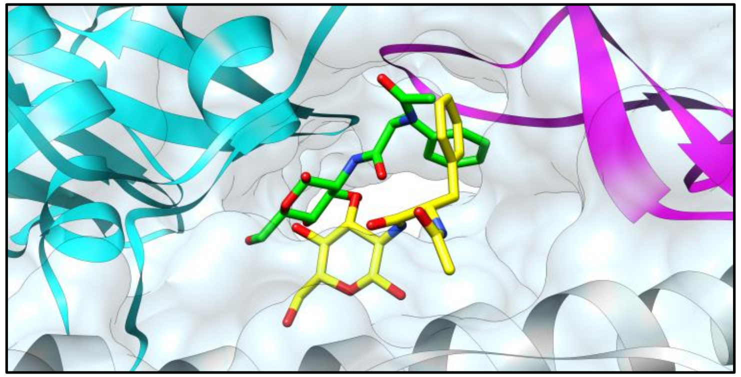

Figure S3. Overlay of the initial (yellow) and final (green) poses from the MD simulation.

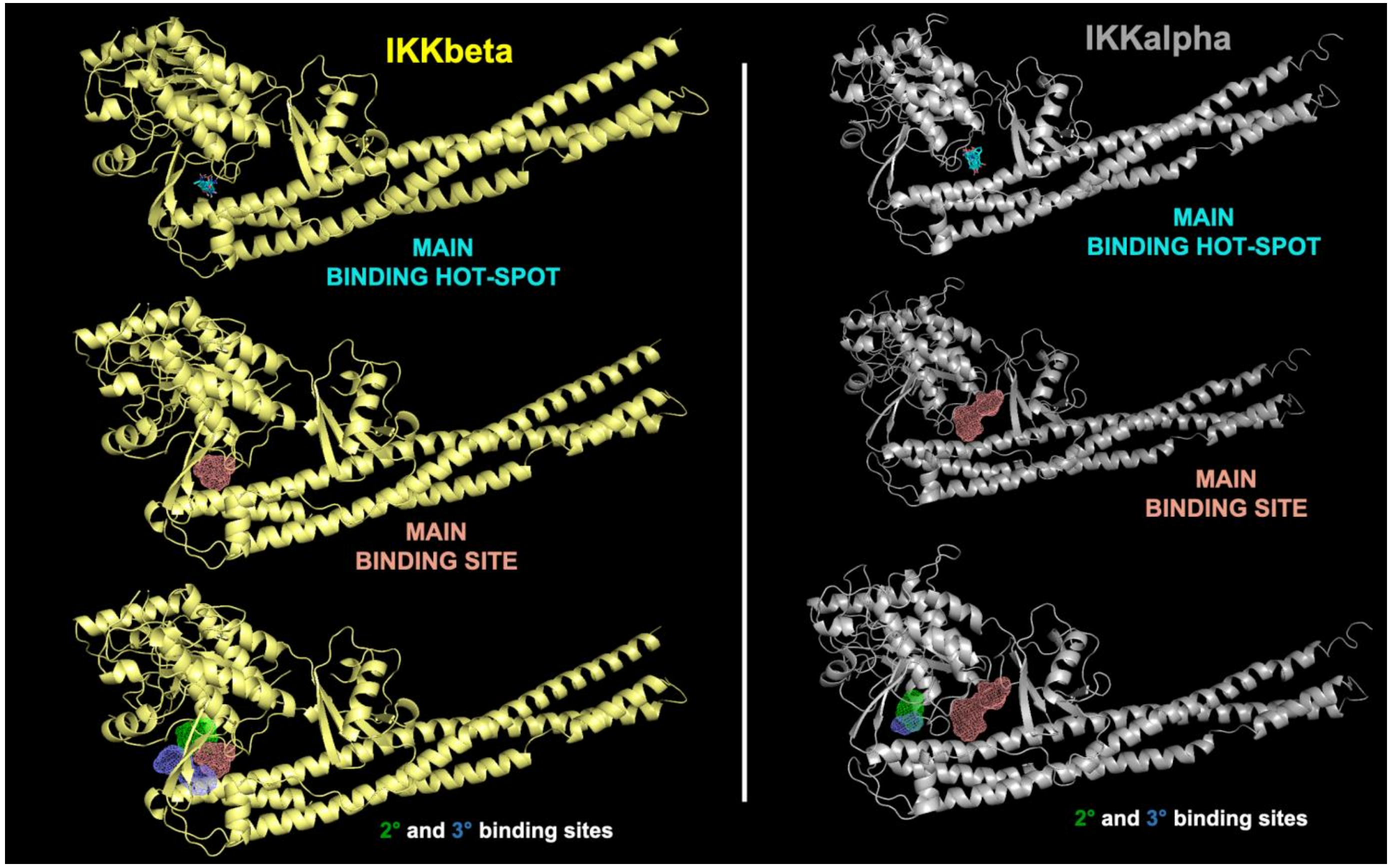

Figure S4. Comparison of FTMap binding sites prediction performed on IKKα over IKKβ: although the two proteins are very similar (almost 60% sequence identity), they present a different position of “druggable” binding sites.
